# *In Vitro* Characterization and Real-Time Label-Free Assessment of the Interaction of Chitosan-Coated Niosomes with Intestinal Cellular Monolayers

**DOI:** 10.1021/acs.langmuir.3c00728

**Published:** 2023-06-02

**Authors:** Elena Scurti, João Pedro Martins, Christian Celia, Paola Palumbo, Francesca Lombardi, Dalila Iannotta, Luisa Di Marzio, Hélder A. Santos, Tapani Viitala

**Affiliations:** † Drug Research Program, Division of Pharmaceutical Chemistry and Technology, Faculty of Pharmacy, University of Helsinki, Helsinki 00014, Finland; ‡ Department of Pharmacy, University of Chieti − Pescara “G. d’Annunzio”, Chieti 66100, Italy; § Laboratory of Drug Targets Histopathology, Institute of Cardiology, Lithuanian University of Health Sciences, A. Mickeviciaus g. 9, Kaunas 44307, Lithuania; ∥ Department of Life, Health & Environmental Sciences, University of L’Aquila, L’Aquila 67100, Italy; ⊥ Australian Institute for Bioengineering and Nanotechnology, The University of Queensland, Brisbane, QLD 4072, Australia; # Department of Biomedical Engineering, University Medical Center Groningen, University of Groningen, Ant. Deusinglaan 1, Groningen 9713, The Netherlands; % W.J. Kolff Institute for Biomedical Engineering and Materials Science, University Medical Center Groningen, University of Groningen, Ant. Deusinglaan 1, Groningen 9713 The Netherlands; $ Pharmaceutical Sciences Laboratory, Faculty of Science and Engineering, Åbo Akademi University, Turku 20520, Finland

## Abstract

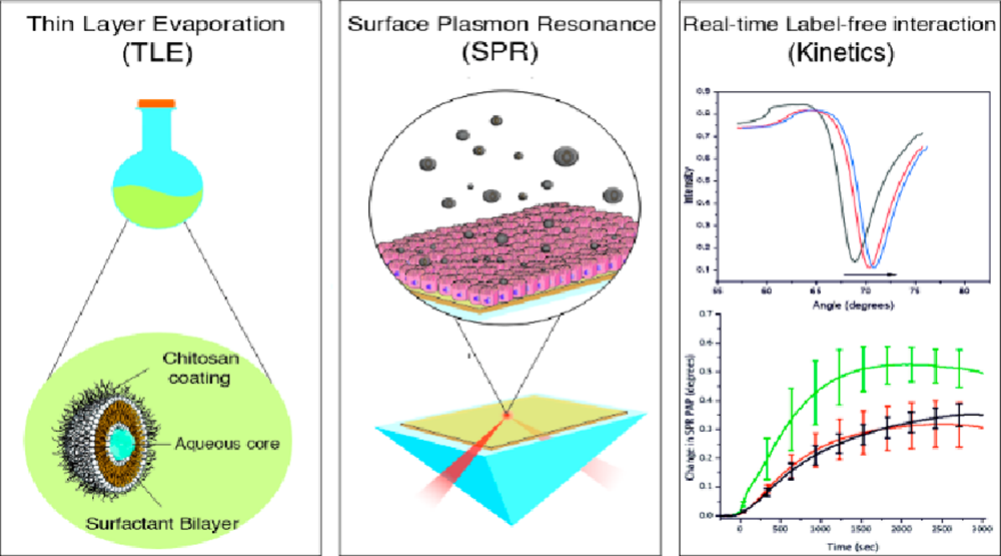

*In vitro* cell-based characterization methods of nanoparticles are generally static and require the use of secondary analysis techniques and labeling agents. In this study, bare niosomes and chitosan-coated niosomes (chitosomes) and their interactions with intestinal cells are studied under dynamic conditions and without fluorescent probes, using surface plasmon resonance (SPR)-based cell sensing. Niosomes and chitosomes were synthesized by using Tween 20 and cholesterol in a 15 mM:15 mM ratio and then characterized by dynamic light scattering (DLS). DLS analysis demonstrated that bare niosomes had average sizes of ∼125 nm, polydispersity index (PDI) below 0.2, and a negative zeta (ζ)-potential of −35.6 mV. In turn, chitosomes had increased sizes up to ∼180 nm, with a PDI of 0.2–0.3 and a highly positive ζ-potential of +57.9 mV. The viability of HT29-MTX, Caco-2, and Caco-2/HT29-MTX cocultured cells showed that both niosomes and chitosomes are cytocompatible up to concentrations of 31.6 μg/mL for at least 240 min. SPR analysis demonstrated that chitosomes interact more efficiently with HT29-MTX, Caco-2, and Caco-2/HT29-MTX cocultures compared to bare niosomes. The resulting SPR measurements were further supported by confocal microscopy and flow cytometry studies, which demonstrated that this method is a useful complementary or even alternative tool to directly characterize the interactions between niosomes and *in vitro* cell models in label-free and real-time conditions.

## Introduction

In nanomedicine nanoparticles are modified with fluorescent probes or radiotracers when studying their interactions with cells/tissues or after *in vivo* administration.^1,2^ Fluorescent labels are also frequently used *in vitro* for cell assays that are often static, require secondary detection techniques, and lack the ability to monitor nanoparticle–cell interactions and uptake in real time. On one hand, the adsorption and/or conjugation of fluorescent probes or radiotracers to nanoparticles can modify their cellular interactions and uptake, as these molecules can alter their different physicochemical characteristics.^3^ On the other hand, the use of fluorescent probes to label cells or cell organelles can interfere with cell behavior, which can also lead to misleading interpretations when analyzing nanoparticle–cell interactions. Consequently, promising *in vitro* results might often not be followed by corresponding success *in vivo*, which hampers an efficient translation of nanoparticle-based drug delivery systems from bench to bedside.

Surface plasmon resonance (SPR)-based cell sensing is an innovative biosensing technique, which is emerging as a promising complementary or alternative tool to traditional characterization methods currently in use to study *in vitro* interactions between nanoparticles and cells or cellular membranes.^4^ The optical SPR phenomenon, extensively described in the literature,^5^ occurs at a certain angle of the incident laser (SPR angle), and it is proportional to the change in refractive index (*n*) and thickness (*d*) of the layer in proximity of the metal sensor surface.^6^ In this technique, the interaction of, for example, nanoparticles with cells is represented by shifts in the SPR angle (θ) which are caused by morphological or intracellular redistribution of material within the cells seeded on top of the metal sensor surface when they are exposed to nanoparticles. As a development of the traditional SPR technique, limited to biomolecular interaction assays,^7^ due to its restricted angular detection range,^7,8^ a wide angular scanning SPR can utilize sensing layers with thicknesses up to several micrometers, therefore enabling the monitoring of drug or nanoparticle interactions with cell monolayers in real time, without using labels, and above all, keeping both the drug or nanoparticles and the cells intact.^5,9^

It has recently been demonstrated that wide angular scanning SPR is a convenient and easily adaptable technique to study biophysical properties of lipid-based nanoparticles, such as liposomes, after their interaction with human plasma^10^ or the vitreous.^11^ However, although liposomes are convenient and easily modifiable drug delivery systems, they have several challenges compared to other nanocarriers, such as niosomes, when considering oral drug delivery applications. The preparation of liposomes is rather time-consuming, as it requires the combination of several different processes, making their manufacturing and scale-up challenging. Therefore, achieving stable formulations with a long shelf life becomes expensive.^12^ Moreover, oral administration of liposomes, for instance, causes the degradation of the lipid bilayer and leakage of payloads due to the acidic pH and high enzymatic activity of the gastrointestinal tract (GIT).^13^

To overcome issues related to lipid degradation, nonionic surfactant vesicles, or niosomes, have emerged as alternative drug delivery systems.^14^ Niosomes can be synthesized using amphiphilic surfactants (e.g., Tween or SPAN)^15,16^ that can self-assemble into supramolecular structures and form bilayer structures like liposomes. Furthermore, niosomes have a lower cost of production, higher shelf life, and storage stability compared to liposomes.^15^

In this study, bare niosomes and chitosan-coated niosomes (chitosomes) were prepared and characterized as potential drug delivery nanocarriers for oral drug administration. Bare niosomes were synthesized by using the thin layer evaporation method with Tween 20 and cholesterol at equivalent molar concentrations, while chitosomes were obtained by an additional coating with chitosan. Chitosan is a positively charged deacetylated polymer of chitin, and it was used due to its mucoadhesive properties,^17,18^ which increase retention time, permeability, or even uptake of drugs in the intestinal microenvironment. Niosomes and chitosomes were characterized for their hydrodynamic diameter (*Z*-average), polydispersity index (PDI), and zeta (ζ)-potential by dynamic light scattering (DLS) and electrophoretic mobility measurements, and their morphology was studied by transmission electron microscopy (TEM). Stability studies were performed in cell culture medium and buffer solutions that resemble the pH variations found in the GIT. Real-time label-free interaction studies between niosomes or chitosomes and *in vitro* cell models of the human intestinal epithelium were performed with a wide angular scanning SPR by culturing HT29 treated with the methotrexate (HT29-MTX), Caco-2, and Caco-2/HT29-MTX (90:10 ratio) cells on SPR gold sensors. Complementary studies for the viability of mono- and cocultured cells against different niosome and chitosome concentrations and incubation times were evaluated with the cell counting kit (CCK-8) assay, while niosome and chitosome interactions with different cells were further investigated by confocal microscopy and flow cytometry.

## Experimental Section

### 2.1 Materials

Tween 20, Sephadex G75 glass columns, low molecular weight chitosan (50–190 kDa, acetylation degree of 75–85%), 2-(4-(2-hydroxyethyl)piperazin-1-yl)ethanesulfonic acid (HEPES), CCK-8, paraformaldehyde (PFA), 30% (v/v) hydrogen peroxide, and 30% (v/v) ammonia hydroxide were obtained from Sigma-Aldrich (St. Louis, MO). Cholesterol was obtained from Acros Organics BVBA (Geel, Belgium). CellMask DeepRed, Hank’s balanced salt solution (10× HBSS), Trypan Blue staining 0.4% (v/v), and fetal bovine serum (FBS) were purchased from Life Technologies Gibco (Waltham, MA). Phosphate buffered saline (10× PBS), Dulbecco’s modified Eagle’s medium (DMEM) (high glucose), nonessential amino acids (NEEA), l-glutamine 200 mM, penicillin (100 IU/mL), streptomycin (100 mg/mL), and trypsin 2.5% were obtained from HyClone, GE Healthcare Lifesciences (Logan, UT). An 8-well Nunc Lab-Tek II Chamber Slide was obtained from ThermoFisher Scientific (New York, NY). Lissamine rhodamine B 1,2-dihexadecanoyl-*sn*-glycero-3-phosphoethanolamine triethylammonium salt (rhodamine B-DHPE) was purchased from Invitrogen (New York, NY). Vectashield antifade mounting medium containing 4′,6-diamidino-2-phenylindole dihydrochloride (DAPI) was purchased from Vector Laboratories, Inc. (Burlingame, CA). 12-BD Falcon cell culture inserts (pore size 0.4 μm, growth area 0.9 cm^2^) were obtained from Becton Dickinson (Milan, Italy). Tissue culture flasks and 96-well plates were obtained from Corning Inc. (New York, NY). Caco-2 (human colon adenocarcinoma) cells were purchased from American Type Culture Collection (ATCC; Manassas, VA). Human goblet-like HT29-MTX was kindly provided by Dr. T. Lesuffleur from INSERM U178 (Villejuif, France). Ethanol 99.5% (v/v) was purchased form Altia Oyi (Helsinki, Finland). Gold-coated SPR sensors were provided by Bionavis Ltd. (Tampere, Finland).

### 2.2 Preparation and Purification of Bare Niosomes

The synthesis of bare niosomes was performed by the thin layer evaporation method,^16^ using an equimolar concentration of Tween 20 and cholesterol (15 mM:15 mM). The mixture was then dissolved in a chloroform and methanol organic mixture (3:1; v/v), and the resulting lipid film was obtained by removing organic solvent with a Rotavapor, Buchi Interface I-100 (Buchi, Switzerland), at room temperature (RT) and gradually decreasing vacuum from 500 to 20 mbar. The residual organic solvent was further removed under vacuum at RT and 20 mbar. The resulting lipid film was then hydrated by adding an aqueous solution containing 10 mM of HEPES buffer in deionized water. The surfactant dispersion was then mechanically stirred for 5 min and sonicated for 10 min at 60 °C with a Hielscher probe sonicator UP200H (Teltow, Germany) equipped with an exponential microprobe operating at 24 kHz and an amplitude of 60%.

Vesicle suspensions were purified by size exclusion chromatography on a Sephadex G75 using 10 mM of HEPES buffer solution at pH 7.4 as the mobile phase, as previously reported.^19^

Fluorescent-labeled niosomes were obtained by adding 0.1 mL of a Rhodamine B-DHPE suspension (2 mg/mL dissolved in ethanol) into the organic phase. The resulting fluorescent labeled niosomes were synthesized and purified with the method mentioned above.

### 2.3 Quantification of the Self-Assembly of Surfactants into Bare Niosomes

The assembly of surfactants into niosomes was quantified using a previously reported colorimetric method.^16^ Briefly, unknown volumes of niosomes, diluted in 10 mM of HEPES (3 mL) at pH 7.4, previously purified through a Sephadex-G75 column, were treated with a cobalt thiocyanate solution (3 mL) and then extracted using dichloromethane (3 mL). The concentration of self-assembled surfactants was evaluated by measuring the absorbance of the organic phase after the extraction with a Cary 50 Scan spectrophotometer (Varian Inc. Corporate; Palo Alto, CA) at 620 nm. Samples were quantified using an external linear calibration plot of standards obtained with different surfactant concentrations (0.063–1.0 mg/mL) (Figure S1). The percentage of self-assembled niosomes represents the ratio of surfactant (w/w), which is compared to the total amount component added to the solution during the preparation procedure.

### 2.4 Preparation of Chitosan-Coated Niosomes (Chitosomes)

After purification of bare niosomes, chitosan was dissolved in a 0.2 M acetic acid solution under magnetic stirring at RT, to a final concentration of 0.2% (w/v). Bare niosomes with an initial concentration of 0.95 mg/mL in 10 mM of HEPES (pH 7.4) were added dropwise to an equal volume of chitosan solution under continuous magnetic stirring. After this, the mixture was stabilized by incubation for 1 h at 10 °C. The ratios of chitosan/Tween 20 tested for the preparation of chitosomes were 0.2, 0.4, 0.6, 0.8, 1, and 1.2 (w/w). The final pH of the chitosome solution was maintained at pH 4.5, which is equivalent to the pH of the chitosan solution.

### 2.5 Physicochemical Characterization of Bare Niosomes and Chitosomes

The particle size (*Z*-average), PDI and ζ-potential of bare niosomes and chitosomes were determined by DLS and electrophoretic mobility with a Zetasizer Ultra instrument (Malvern Panalytical; Malvern, UK). Measurements were conducted at 25 °C, and samples were loaded in disposable cuvettes. Bare niosomes and chitosomes were diluted 1:20 (v/v) in deionized water and prefiltered with polypropylene membranes (pore size 0.22 μm; Whatman Inc., Clifton, NJ) prior to size and ζ-potential measurements to avoid multiscattering phenomena. The following parameters were used for size and PDI measurements: real refractive index 1.59, imaginary refractive index 0.0, medium refractive index 1.330, medium viscosity 1.0 mPa s, and medium dielectric constant 80.4; while the ζ-potential determinations were performed by using a Smoluchowsky constant *F* (Ka) of 1.5 with a He/Ne laser Doppler anemometry (633 nm) and a nominal power of 5.0 mW.^20^

The stability of five different concentrations of chitosomes was tested in buffer solutions mimicking gastrointestinal-tract (the GIT) conditions (pH 1.2, 6.5, and 7.4) by measuring their particle size, PDI, and ζ-potential for up to 240 min. Samples were diluted to concentrations of 11.9–190 μg/mL and incubated for varying periods of time under magnetic stirring in the buffer solutions. The solution at pH 1.2 was prepared by adding 25 mL of NaCl (6.86 × 10^–2^ M), 2.5 mL of HCl (1.2 M), and 47.5 mL of deionized water. The solution at pH 6.5 was prepared by adding 5 mL of NaOH (0.2 M), 12.5 mL of NaH_2_PO_4_ (0.2 M), and 32.5 mL of deionized water. The solution at pH 7.4 was prepared by adding 7.5 mL of NaOH, 12.5 mL of NaH_2_PO_4_, and 30 mL of deionized water. All experiments were performed in triplicates.

TEM was used to study the morphology of bare niosomes and chitosomes, as previously reported.^21^ For this purpose, copper-coated grids were covered with 10 μL samples, blotted away after 10 min, and left to dry overnight at RT before imaging at 80 kV, in a vacuum, using a JEOL JEM-1400 (Tokyo, Japan).

### 2.6 Cell Lines and Culture Conditions

Caco-2 (passages #34–40) and HT29-MTX (passages #32–40) cells were cultured separately in tissue culture flasks (Corning Inc., New York, NY) in DMEM medium (high glucose) enriched with 10% (v/v) FBS, 1% (v/v) l-glutamine, 1% (v/v) NEEA, and a 1% (v/v) antibiotic–antimitotic mixture (final concentration of 100 IU/mL penicillin and 100 IU/mL streptomycin). The cells were kept in an incubator (16 BB gas, Heraeus Instruments GmbH, Hanau, Germany) at 37 °C and 5% CO_2_ in a water-saturated atmosphere. The culture medium was replaced every other day. The subculturing procedure was performed when the cells reached 70–80% confluency. For this purpose, cells were washed with a solution of PBS–ethylenediaminetetraacetic acid (EDTA), then incubated with trypsin 0.25% (v/v) in PBS–EDTA for 4 min, to promote detachment from the flask, centrifuged, dispersed into fresh growing medium, and transferred to a new flask.

### 2.7 Cell Viability Tests

Viability tests were performed on monocultures of Caco-2 and HT29-MTX cells and cocultures of Caco-2/HT29-MTX (90:10 ratio) using a CCK-8 kit, according to the manufacturer’s instructions. Briefly, the cells were seeded into 96-well plates at a density of 1 × 10^5^ cells/cm^2^, incubated overnight with 100 μL of culture medium, and then treated with chitosomes or free chitosan dispersed in DMEM without FBS supplemented with 10 mM of HEPES at pH 6.5 at the following concentrations: 11.9, 23.5, 31.6, 47.5, 95, and 190 μg/mL. As control, the cells were incubated with DMEM without FBS supplemented with 10 mM of HEPES at pH 6.5. Additional controls consisted of incubating the cells with the lowest (i.e., 11.9 μg/mL) and highest concentration (i.e., 190 μg/mL) of bare niosomes dispersed in the same incubation medium. 10 μL of CCK-8 reagent was added to each well and incubated at 37 °C for 2 h. The absorbance was recorded at a wavelength of 450 nm using a Bio-Rad microplate reader (Hercules, CA). Results are calculated as optical density (O.D.) and then converted into cell viability percentage. The experiment was conducted in triplicates, and the results are presented as mean ± SD (*n* = 3).

### 2.8 Cell Immobilization on SPR Gold Sensor Slides

Before seeding, the gold SPR sensors were cleaned by immersion for at least 10 min in a boiling solution of 30% (v/v) ammonia hydroxide, 30% (v/v) hydrogen peroxide, and deionized water at a ratio of 1:1:5 (v/v/v). Sensors were then rinsed with deionized water and dried under nitrogen flux. Gold sensors were sprayed with 70% (v/v) ethanol before cell seeding and then dried for at least 30 min. The sensors were individually placed in 8.8 cm^2^ Petri dishes. The cell density was optimized for each cell line. Caco-2 and Caco-2/HT29-MTX (90:10 ratio) were seeded at a concentration of 1 × 10^5^ cells/cm^2^, whereas HT29-MTX cells were seeded at a concentration of 2.27 × 10^5^ cells/cm^2^. After seeding, mono- and cocultures were incubated at 37 °C in 5% CO_2_ for periods of 3–4 days for HT29-MTX and 8, 15, and 20 days for Caco-2 and cocultures.

### 2.9 SPR Measurements

SPR experiments were performed using a wide angular scanning MP-SPR Navi 200 OTSO instrument (angular scan range 40°–78°; Bionavis Ltd., Tampere, Finland) equipped with four flow channels and an external peristaltic pump for controlling the running buffer flow. The flow channels and optical system were preheated to 37 °C, and the fluidic system was primed with the running buffer (DMEM without FBS, supplemented with 10 mM of HEPES, pH 6.5). The formation of a confluent cell monolayer was verified by observing the sensors under an optical microscope before each experiment. Before loading of the sensor slide into the SPR sensor holder, the glass side of the gold sensor was wiped with 99% (v/v) ethanol to remove any medium residues or impurities. Afterward, the sensor holder was placed into the instrument, and the pump was immediately operated with a flow speed of 20 μL/min. The full SPR reflectance spectra between 40° and 78° were recorded as a function of time until a stable baseline was achieved in the SPR angular response. Hereafter, the angular range was changed to 58° and 76°, with a scanning time of approximately 2 s. The SPR measurements were performed using a laser wavelength of 670 nm. Before and between each measurement, the cells were allowed to stabilize for at least 30 min. The main SPR angle for the cell-free sensors in the running buffer was between 69° and 70°. For cell-seeded sensors, the main SPR angle increased to 72.5°. Samples were injected 30 min after the stabilization of the SPR angular response. After this, samples were injected through the flow channels for up to ∼50 min. Bare niosomes and chitosomes (initial concentration of 0.95 mg/mL) were diluted in the running buffer to predetermined concentrations. The chitosan stock solution from which control samples of free chitosan were prepared was made dissolving chitosan (218 kDa) in 0.5% (v/v) acetic acid to a final concentration of 0.95 mg/mL. The chitosan control solutions were prepared by diluting the stock solution to the desired concentrations (11.9–47.5 μg/mL) with running buffer.

### 2.10 Cell Interaction Studies of Bare Niosomes and Chitosomes

Confocal laser scanning microscopy (CLSM) and flow cytometry were used to further evaluate the interaction between DHPE-rhodamine B-labeled bare niosomes and chitosomes with cells. The fluorescence intensity of DHPE-Rhodamine B niosomes was quantified with a Varioskan LUX multimode microplate reader (Thermo Fisher Scientific, Waltham, MA). For the CLSM studies, Caco-2/HT29-MTX cocultures (90:10 ratio) were seeded into a 8-well Nunc Lab-Tek II Chamber Slide (Thermo Fisher Scientific) at a concentration of 2 × 10^5^ cells/mL and maintained in a humidified atmosphere at 37 °C and 5% CO_2_ for 24 h. Afterward, cells were washed with HBSS-HEPES (pH 7.4) and incubated with bare niosomes (23.8 μg/mL) and chitosomes (23.8 μg/mL) dispersed in the SPR running buffer (10 mM of HEPES in DMEM, without FBS, pH 6.5) for 3 h at 37 °C. Cells incubated with plain running buffer served as control. After the incubation, the medium was discarded, and cells were washed with a prewarmed solution of HBSS-HEPES (pH 7.4) to remove noninteracting bare niosomes or chitosomes. All the chambers were fixed with 200 μL of 4% (v/v) PFA for 15 min, and the nuclei were stained with 100 μL of DAPI. Cells were observed with a Leica SP5 II HCS-A CLSM (Leica Microsystems, Wetzlar, Germany), as previously reported.^22^

For flow cytometry analysis, Caco-2/HT29-MTX cocultures (90:10 ratio) were seeded at a density of 1 × 10^5^ cells/insert in 12-well Transwell permeable supports (pore size: 0.4 μm; surface area: 1.1 cm^2^; Corning Inc., New York, NY) and incubated with DMEM supplemented with FBS and 10 mM of HEPES at pH 7.4, for 16–21 days at 37 °C, 95% relative humidity, and 5% CO_2_, thus allowing for cellular differentiation and the formation of a polarized cell layer. The medium was replaced every 2 days with 1.0 mL of fresh medium in the apical (AP) chamber and 1.5 mL in the basolateral (BL) chamber. The integrity of the cell monolayer was evaluated by monitoring the transepithelial electrical resistance (TEER) every other day, using a Millicell ERS-2 system (Millipore Corporation, Bedford, MA), equipped with an STX01 electrode (World Precision Instruments, Sarasota, FL), as previously described.^19,23^ Inserts with TEER values between 600 and 800 Ω·cm^2^ were used for further studies.^19,23^ Then, medium in the AP side of the Transwells was removed, and Caco-2/HT29-MTX cocultures on the AP side were incubated with DMEM without FBS, supplemented with 10 mM of HEPES at pH 6.5 (control), bare niosomes (23.8 μg/mL), and chitosomes (23.8 μg/mL) at different incubation times (25–240 min). Caco-2/HT29-MTX monolayers were then harvested using trypsin-EDTA, centrifuged (400*g*, 10 min, 4 °C), washed twice with 2 mL of PBS, and then redispersed into 1 mL of PBS. To study the uptake of bare niosomes and chitosomes, cells collected from each insert were counted, suspended to a final concentration of 10^6^ cell/mL, and analyzed by flow cytometry. To distinguish the associated or bound bare niosomes/chitosomes from the internalized bare niosomes/chitosomes, cells were treated with trypan blue, as previously reported.^24^ Data from 20000 events per sample were collected, and the fluorescence intensity was measured and analyzed in the FL2 channel (λ_ex_ = 488 nm and λ_em_ = 625 nm) by a FACSCalibur flow cytometer equipped with CellQuest software (Becton Dickinson, San Jose, CA) for data acquisition. The bare niosome or chitosome uptake monitored by DHPE-Rhodamine B fluorescence is shown in histogram mode on a logarithmic scale. The resulting graphs showed the percentage of fluorescent-positive cells. The fluorescence intensity of untreated Caco-2/HT29-MTX cells was used as blank.

## Results and Discussion

### 3.1 Physicochemical Characterization

The prescreenings to optimize the synthesis of chitosomes were performed by measuring the ζ-potential of native, undiluted samples with increasing ratios of chitosan/Tween 20 (w/w). The ζ-potential of bare niosomes was highly negatively charged, while the addition of chitosan to bare niosomes reversed the surface charge to positive. This result suggests the successful formation of chitosomes (Figure S2). The surface charge consistently increased when using chitosan/Tween 20 ratios up to 1:1 (w/w) and remained practically constant for higher ratios. For this reason, the chitosan/Tween 20 ratio of 1:1 (w/w) was chosen for further experiments.

**Figure 1:**
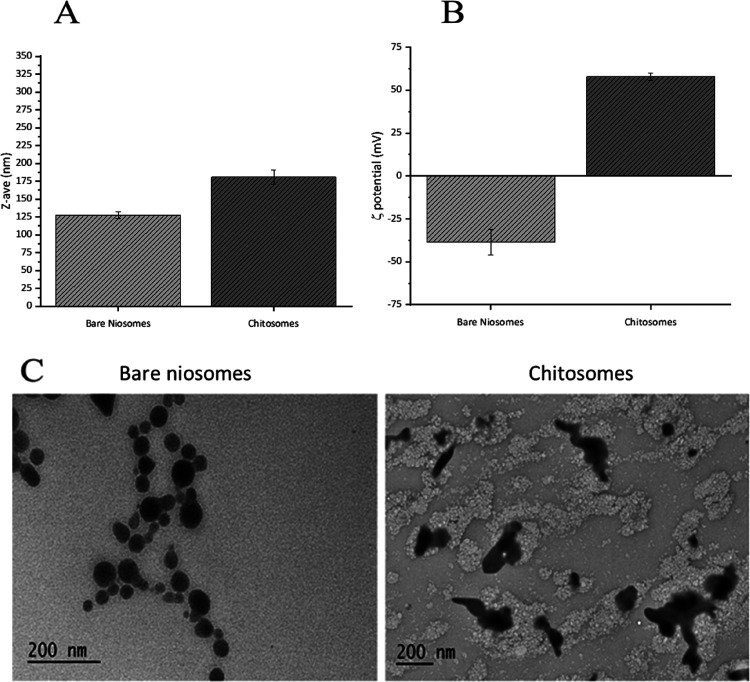
Physicochemical characterization of the prepared nanoparticles. Hydrodynamic diameter (A), ζ-potential (B), and TEM images (C) of bare niosomes and chitosomes. Dark areas in the TEM images represent the core structures of niosomes, while the gray irregular shapes surrounding the dark areas in the case of chitosomes represent chitosan. Samples were diluted before the analysis in 1:20 (v/v) Milli-Q water and prefiltered with polypropylene membranes (pore size 0.22 μm) to avoid multiscattering phenomena. Results of the DLS and ζ-potential measurements are presented as mean ± SD (*n* = 3).

Bare niosomes and the optimized chitosomes were further characterized for their size, PDI, and ζ-potential after diluting the samples 1:20 (v/v) in deionized water and prefiltering with polypropylene membranes (Figure 1A,B). Results showed that bare niosomes have an average size of 125 ± 5 nm (Figure 1A) and a narrow size distribution with a PDI of 0.11 ± 0.02, while chitosomes have an average size of 180 ± 10 nm (Figure 1A) and are slightly more widely distributed, with a PDI of 0.23 ± 0.03. The increase observed in the average sizes and PDI of chitosomes compared to bare niosomes is because chitosan coats the surface of niosomes, thus increasing the hydrodynamic radius of the colloidal nanoparticles. Moreover, the presence of free/nanoaggregated chitosan in equilibrium with chitosomes and dispersed in the colloidal suspension may also lead to an increased PDI. The ζ-potential of bare niosomes was highly negative (−35.6 ± 7.4 mV; Figure 1B) due to the presence of polyoxyethylene chains, which have similar structure and biopharmaceutical properties to poly(ethylene glycol)s.^19,23^ These chains, bound to the sorbitan ring, orientate water molecules to form hydrogen bonds, in which oxygens of Tween 20 are “hydrogen-bond donors”.^25^ Furthermore, surfactants with a high hydrophilic–lipophilic balance (HLB), like Tweens, possess a higher surface energy, resulting in greater stability of the dispersion and a shift toward negative values of the ζ-potential.^26,27^ The ζ-potential of chitosomes, in turn, was highly positive (+57.9 ± 2.1 mV; Figure 1B), which is a consequence of the presence of positively charged chitosan adsorbed on the surface of the niosomes.

The TEM analysis of the nanocarriers showed that bare niosomes had a spherical shape with regular outlines (Figure 1C, left side), whereas chitosomes had a highly irregular shape (Figure 1C, right side). This effect can depend on the presence of chitosan adsorbed onto the surface of niosomes. Moreover, free chitosan may form aggregates during the drying process associated with the preparation of TEM grids. Free chitosan is dispersed in the background of the TEM images (Figure 1C, right side), and it can cause a dynamic equilibrium between the “aggregated form” of the biopolymer and its “adsorbed form” around the niosomes. In fact, chitosan spontaneously aggregates, thus making nano- and microparticles when diluted in aqueous solution, and its aggregation process strictly depends on the chitosan molecular weight, the degree of acetylation, the pH of the solution affecting the “protonated–deprotonated state” of the amine functions, and the nature of the solvent.^25,28−30^ For this reason, the solution containing chitosomes was kept at a pH below the p*K*_a_ of amine groups to maintain the chitosan electrostatically bound to the negatively charged surface of niosomes and to avoid aggregation during the synthesis due to the repulsive forces among positive charges of the biopolymer.^31^

Overall, these results agree with previously reported data, where it was shown that the percentage of self-assembled Tween 20 was ∼40% (w/w).^32^ This value is affected by the critical packaging parameter of Tween 20, which depends on its critical micellar concentration.^33^ Moreover, DLS and TEM data confirmed the successful preparation of niosomes and chitosomes.

### 3.2 Stability Studies

**Figure 2:**
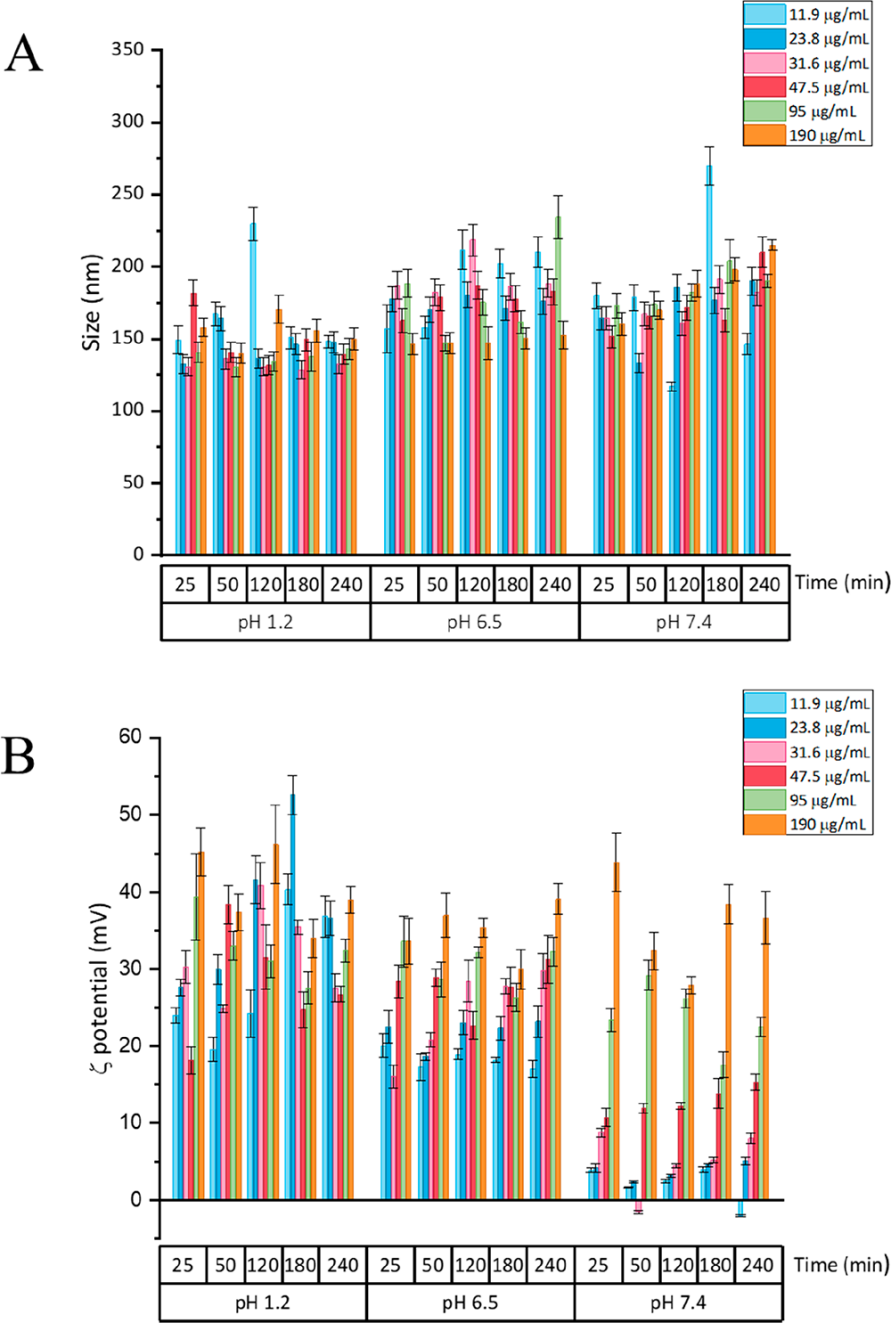
Size (A) and ζ-potential (B) of chitosomes in different buffers mimicking the pH of the GIT (pH 1.2, 6.5, and 7.4) at different concentrations (11.9, 23.8, 31.6, 47.5, 95, and 190 μg/mL) and incubation times (25, 50, 120, 180, and 240 min). Results are presented as mean ± SD (*n* = 3).

Niosomes with the same composition of those prepared herein have previously shown a good colloidal stability, also in simulated gastric fluids.^33,34^ Therefore, we focused on investigating possible changes in the physicochemical properties of chitosomes when incubated with buffers at different pH conditions. Because the chitosomes developed in this study are envisaged for oral administration, the buffers used in this experiment were chosen to mimic the pH variations found throughout the gastrointestinal tract (GIT).^35^ Stability studies were thus performed by measuring average sizes, PDI, and ζ-potential of chitosomes when incubated at different concentrations in buffer solutions at different pH (1.2, 6.5, and 7.4) and for a period of time ranging from 25 to 240 min (Figure 2). Results showed that, overall, the average sizes of chitosomes ranged from 150 to 200 nm at different pH conditions, regardless of their concentration or duration of the incubation, thus demonstrating that chitosomes were stable for an incubation time of up to 240 min (Figure 2A). Moreover, the data suggest that the sizes of the particles at pH 6.5 and 7.4 were slightly larger than those obtained when chitosomes were incubated at pH 1.2. Nonetheless, no particle aggregation was observed, i.e., no drastic increase in particle size was seen, which suggests that the particles are stable in pH conditions mimicking the GIT. The ζ-potential values, in turn, showed more pronounced variations when chitosomes were incubated at different concentrations and pH conditions over time (Figure 2B). At pH 1.2, the ζ-potential of chitosomes varied between +18 and +53 mV, with no clear trend in the behavior of the particles. However, at pH 6.5 and 7.4, the ζ-potential values increased as a function of chitosome concentration. Moreover, the incubation time did not seem to influence these values. This effect was increased when chitosomes were incubated at pH 7.4, with the three lowest particle concentrations showing ζ-potential values equal to or lower than +10 mV and the highest particle concentrations reaching values as high as +44 mV. This may be explained by a lower protonation degree of chitosan at pH 7.4,^31^ which decreases the tendency for chitosan to adsorb and coat the negatively charged niosomes when they were dispersed in smaller concentrations.

Most of the drugs delivered orally are absorbed in the upper intestine, which has a pH of around 6.5. Therefore, in view of the oral use of the particles developed herein and for further *in vitro* cell studies, the size and ζ-potential of bare niosomes and chitosomes at different concentrations were measured after incubation with cell culture medium supplemented with 10 mM of HEPES (pH 6.5) (Figure S3). Results showed that the size of bare niosomes remained between 110 and 130 nm in the cell culture medium with pH 6.5, and their ζ-potential varied between −5 and −8 mV, suggesting that, overall, the samples were stable. Chitosomes also showed a relatively good stability when dispersed in cell culture medium (pH 6.5). In these conditions, the particle size of chitosomes remained around 150 nm, regardless of the particle concentration, while the ζ-potential increased slightly from +2 to +12 mV with increasing particle concentration. The increase in ζ-potential when the chitosome concentration increases follows the same trend as in Figure 2. However, the magnitude of the ζ-potential values for both bare niosomes and chitosomes are smaller in the cell culture medium compared to the values in Figures 1 and 2 because the cell culture medium contains more substances that can screen or shield the charges on the particle surfaces.^36^

### 3.3 Cell Viability Tests

**Figure 3:**
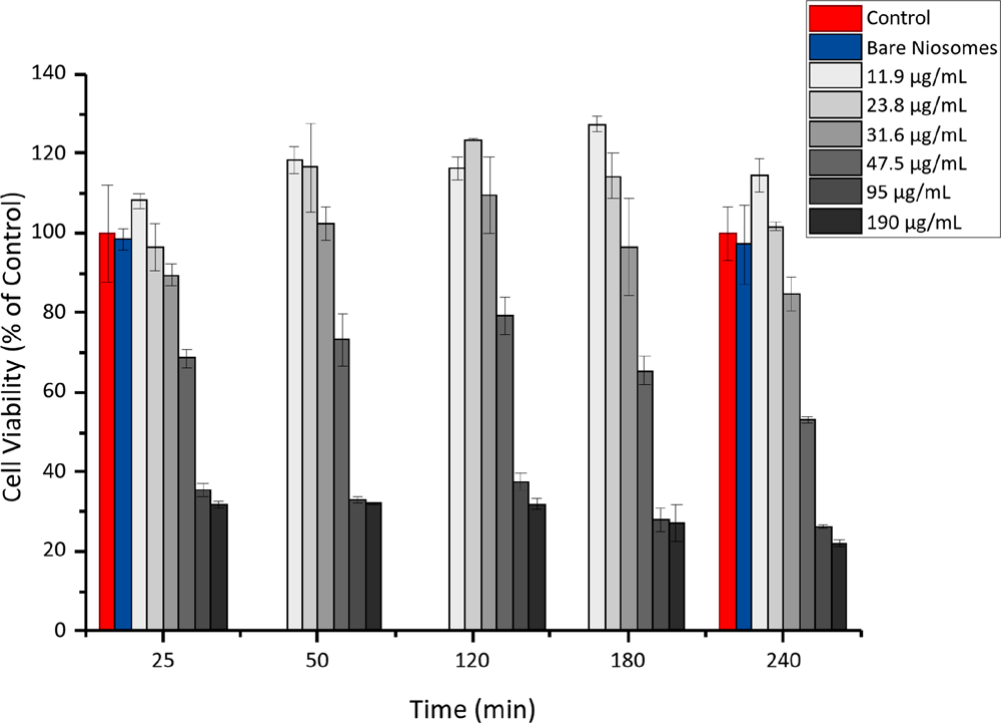
Viability of Caco-2/HT29-MTX cocultures upon exposure to different concentrations of chitosomes (11.9–190 μg/mL) at different incubation times (25–240 min). The red bars represent cells treated with DMEM supplemented with 10 mM of HEPES (pH 6.5), and the blue bars represent bare niosomes at the lowest and highest concentration (25 and 190 μg/mL, respectively). Results are presented as mean ± SD (*n* = 3).

The CCK-8 test was used to study the viability of Caco-2/HT29-MTX cocultures upon exposure to chitosomes at different concentrations and incubation times (25–240 min), dispersed in cell culture medium at pH 6.5. Cells exposed to cell culture medium and to the highest concentration of bare niosomes (i.e., 190 μg/mL) were used as controls, and the viability measurements with the control samples were conducted only at the first and last time points because previous studies have shown that niosomes are nontoxic toward intestinal epithelial cell lines even at higher concentrations than the ones chosen in this work.^16^ Cell viability data show a concentration-dependent effect with the highest chitosome concentrations inducing higher cellular toxicity (Figure 3). Indeed, chitosomes at concentrations of 11.9 and 23.8 μg/mL were not toxic up to 240 min. Conversely, the cell viability percentage decreased upon increasing chitosome concentration above 31.6 μg/mL (Figure 3). In turn, bare niosomes presented cell viabilities of ∼98% for up to 240 min and therefore did not induce any toxicity on these cocultures. Hence, the cytotoxicity observed for the highest concentrations of chitosomes most probably derives from the presence of chitosan in the formulation.^37^ This is also supported by a previous study that has shown a Caco-2 cell viability of about 50% upon exposure to chitosan-coated niosomes at concentrations around 100 μg/mL.^38^ However, the presence of acetate buffer in the highest concentration (i.e., 0.01 M of acetate buffer in the 190 μg/mL chitosomes sample) may also contribute to the decrease in viability through modulation of mitochondrial function, as previously reported for the HT29 cell line.^39^ Cell viability studies were also conducted on HT29-MTX and Caco-2 monocultures under the same incubation conditions (Figure S4). Results for the monocultures agreed with those observed for the cocultures; i.e., cellular toxicity is induced by chitosome concentrations above 31.6 μg/mL.

### 3.4 Real-Time SPR Analyses of Chitosome–Cell Interactions

Because chitosomes are coated with positively charged chitosan, they are expected to interact more strongly with cells than the bare, negatively charged niosomes, especially with the negatively charged mucins present in the mucus layer of HT29-MTX cells.^18^ This hypothesis was demonstrated by preliminary SPR measurements performed with bare niosomes and chitosomes interacting with HT29-MTX cells (Figure S5). The SPR peak angular position (PAP) signal response measured during the interaction of chitosomes with HT29-MTX cells is ca. 10× greater than for bare niosomes with the same concentration. This supports the fact that the positively charged chitosan coating on chitosomes facilitates a drastically stronger interaction with cells than bare, negatively charged niosomes. Hence, we focused here on studying the real-time interaction kinetics of chitosomes with various intestinal *in vitro* cell models, including HT29-MTX, Caco-2 monocultures, and Caco-2/HT29-MTX (90:10 ratio) cocultures. The concentrations of chitosomes for performing the SPR interaction measurements with HT29-MTX cells were chosen based on the cell viability studies as 11.9, 23.8, and 47.5 μg/mL. For Caco-2 and Caco-2/HT29-MTX cocultures, the SPR interaction measurements were performed with only a single chitosome concentration of 23.8 μg/mL because this concentration showed a good cell viability, while allowing for a comparison of SPR PAP responses among all three cell models used.

**Figure 4:**
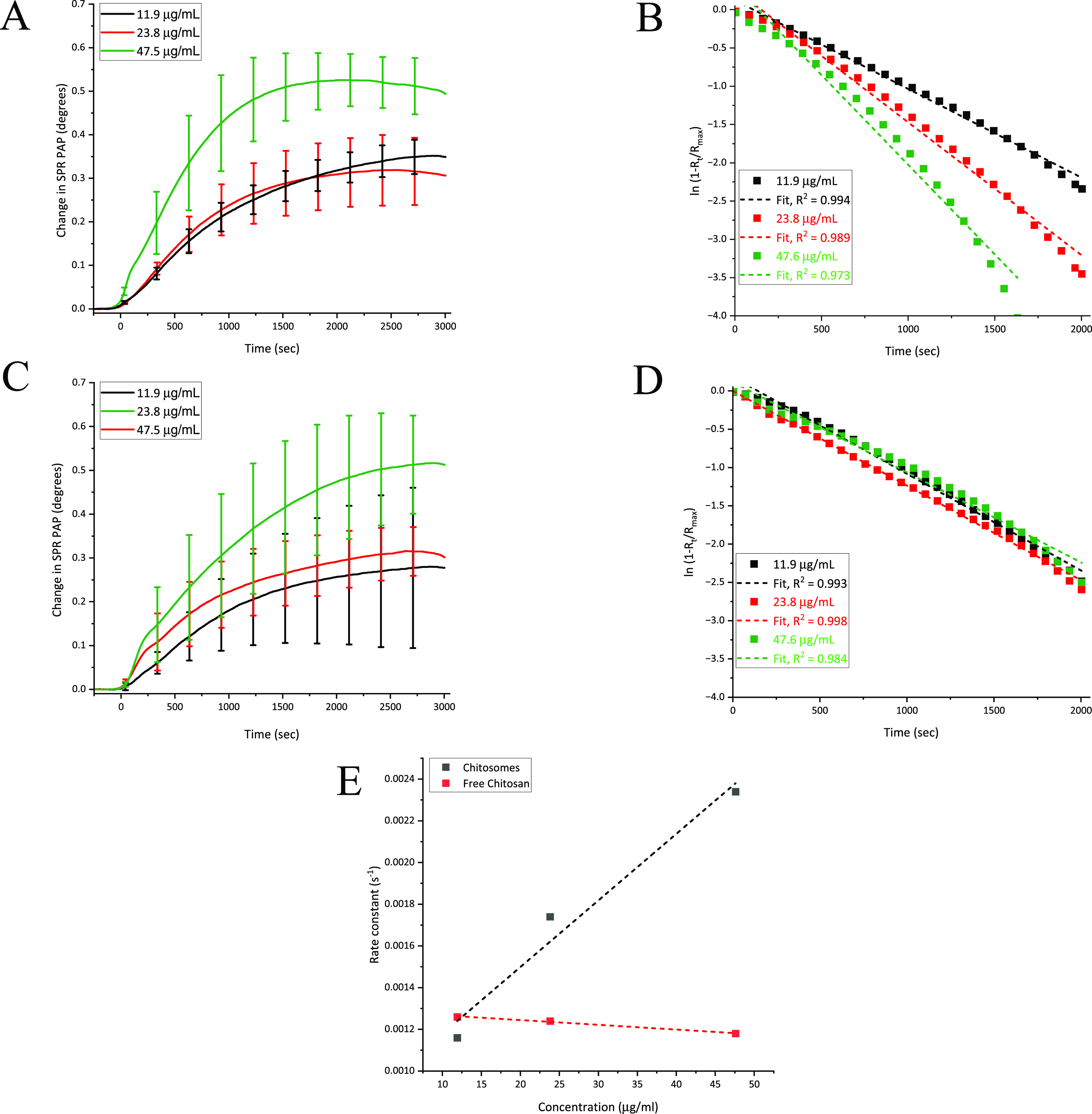
SPR signal responses during interaction of increasing concentration of chitosomes (A) and free chitosan (B) with HT29-MTX cells (*n* = 3). First-order kinetic fits for SPR signal responses during interaction of increasing concentration of chitosomes (C) and free chitosan (D) with HT29-MTX cells. Dashed lines are first-order kinetic fits to data points. First-order interaction rate constants for chitosomes and free chitosan when interacting with HT29-MTX cells, obtained from the linear fitting shown in panels B and D (E). Dashed lines are linear fits according to data points.

Figure 4A shows the SPR PAP responses for increasing concentration of chitosomes when interacting with HT29-MTX monocultures. The SPR PAP responses show a concentration-dependent behavior. This was more noticeable when the SPR PAP responses were fitted with first-order kinetic linear fitting using the equation , where *R* is the SPR response at a certain time during the interaction measurement, *R*_max_ is the maximum SPR response reached during the interaction measurement, and *k* is the interaction rate constant. The fittings showed that the rate of interaction between the chitosomes and HT29-MTX cells clearly increased with increasing chitosome concentration (Figure 4B). Parallel tests with free chitosan samples with concentrations of 11.9, 23.8, and 47.5 μg/mL were performed to clarify whether the rate constant for chitosomes were influenced by free chitosan (Figure 4C). The SPR responses for free chitosan were also fitted with first-order kinetics in the same manner as for chitosomes (Figure 4D).

Figure 4E shows the rate constants as a function of chitosome or free chitosan concentration. Altogether, these results show that the rate constants for chitosomes are not affected by the free chitosan concentration. This is because the rate constants for free chitosan remain practically stable with increasing chitosan concentration, while for the chitosomes, the rate constants increase with increasing chitosome concentrations (Figure 4E). Thus, the SPR PAP responses measured for the chitosomes originate from the interactions of the nanoparticles with the cells, and the influence of the possible remaining free chitosan in chitosome samples would have a negligible effect on their interaction rate constants.

**Figure 5:**
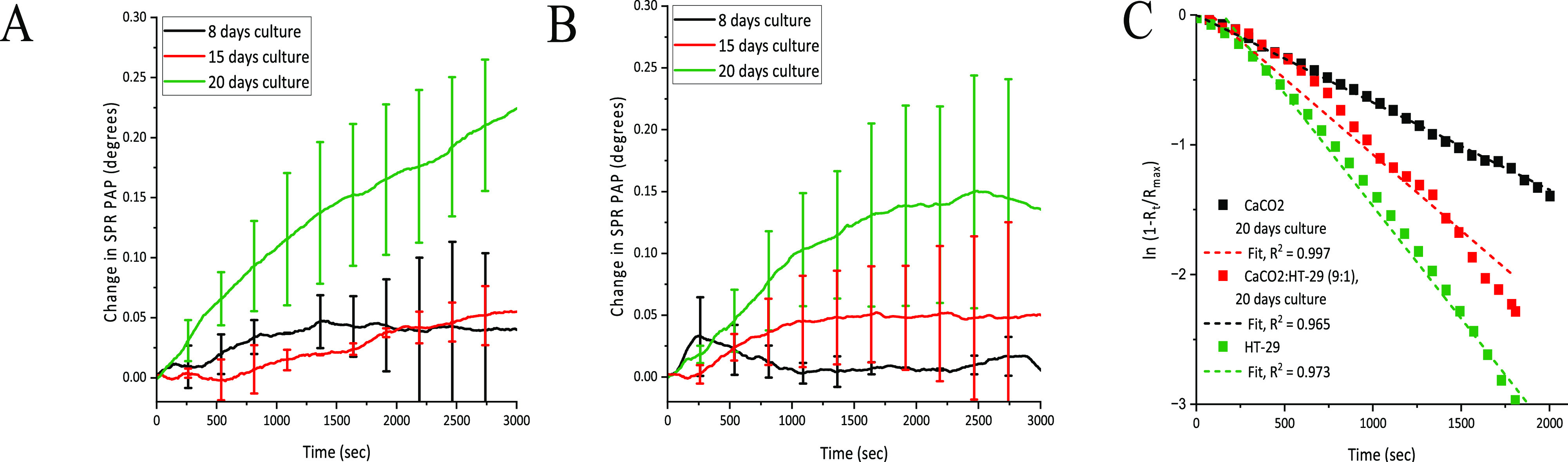
SPR responses during interaction of chitosomes (23.8 μg/mL) with Caco-2 (A) and Caco-2/HT29-MTX cocultured (B) cell monolayers performed after 8 days (black), 15 days (red), and 20 days (green) of seeding on a gold SPR sensor. Results are presented as mean ± Min/Max (*n* = 2). First-order kinetic fits for SPR signal responses during interaction of chitosomes (23.8 μg/mL) with 20 days cultured Caco-2 (black), Caco-2/HT29-MTX cocultured (red), and HT29-MTX cells (green, same as in Figure 4C and included in this graph for comparison) (C). Dashed lines are first-order kinetic fits to data points. The linear fitting was performed as described in Section 3.4.

It is well-known that it can take up to 20 days for Caco-2 cells to form fully polarized cell monolayers with tight junctions.^19,23,40^ Therefore, we chose one concentration of chitosomes (i.e., 23.8 μg/mL) to study how different growth stages of Caco-2 and Caco-2/HT29-MTX cocultured cell monolayers influence the SPR PAP responses for chitosome interactions due to differences in cell monolayer polarization and formation of tight junctions. For this, Caco-2 and Caco-2/HT29-MTX cocultured cell monolayers at different growth stages (i.e., 8, 15, and 20 days) were prepared and allowed to interact with the chitosomes. Figures 5A and 5B show that the SPR PAP responses during chitosome interactions with the Caco-2 and Caco-2/HT29-MTX cocultured cell monolayers increased the longer the cell monolayers had been allowed to mature, which reflects that the chitosomes interact more strongly with cell monolayers with a higher degree of polarization and tight junctions. This indicates that the maturation of the cell monolayer facilitates electrostatic interactions between the positively charged chitosomes and the negatively charged cell surfaces, which becomes more prominent with a higher degree of polarization and tight junctions in the Caco-2 and Caco-2/HT29-MTX cocultured cell monolayers.

Figures 5A and 5B also show that the SPR PAP response during chitosome interactions with Caco-2/HT29-MTX cocultured cell monolayers started to increase earlier, i.e., at 15 days of culturing, compared to Caco-2 for which the SPR PAP response did not increase until 20 days of culturing. Furthermore, it is clearly seen that the presence of HT29-MTX significantly influences the interaction kinetics of chitosomes with the cell monolayers. Figure 5C and Table 1 show that the rate constant for the interaction between chitosomes and the Caco-2/HT29-MTX cocultured cell monolayers is almost twice the rate constant for the interaction between chitosomes and the Caco-2 cell monolayers. Thus, the faster interaction kinetics for Caco-2/HT29-MTX cocultured cell monolayers indicates that the mucus producing HT29-MTX cells facilitate the electrostatic interactions between the chitosomes and the Caco-2/HT29-MTX cocultures. This is also supported by the fact that the interaction kinetics of chitosomes is highest for the plain HT29-MTX cell monolayers compared to Caco-2 and Caco-2/HT29-MTX cocultured cell monolayers (Table 1). The Caco-2/HT29-MTX cocultured cell monolayers were chosen for further studies with confocal microscopy and flow cytometry to determine the interaction and uptake of chitosomes in the intestinal cell model. This was based on the better resemblance of this model to the intestine, as it contains the mucus secreting cells.

**Table 1:** First-Order Interaction Rate Constants for Chitosomes Interacting with Caco-2, Caco-2/HT29-MTX Cocultured, and HT29-MTX Cells Obtained from the Linear Fitting in Figure 5C

cell culture	*k* (s^–1^)
Caco-2	0.67 × 10^–3^
Caco-2/HT29-MTX (90:10 ratio)	1.17 × 10^–3^
HT29-MTX	1.74 × 10^–3^

### 3.5 Interaction and Uptake of Bare Niosomes and Chitosomes in Caco-2/HT29-MTX Cocultures

**Figure 6:**
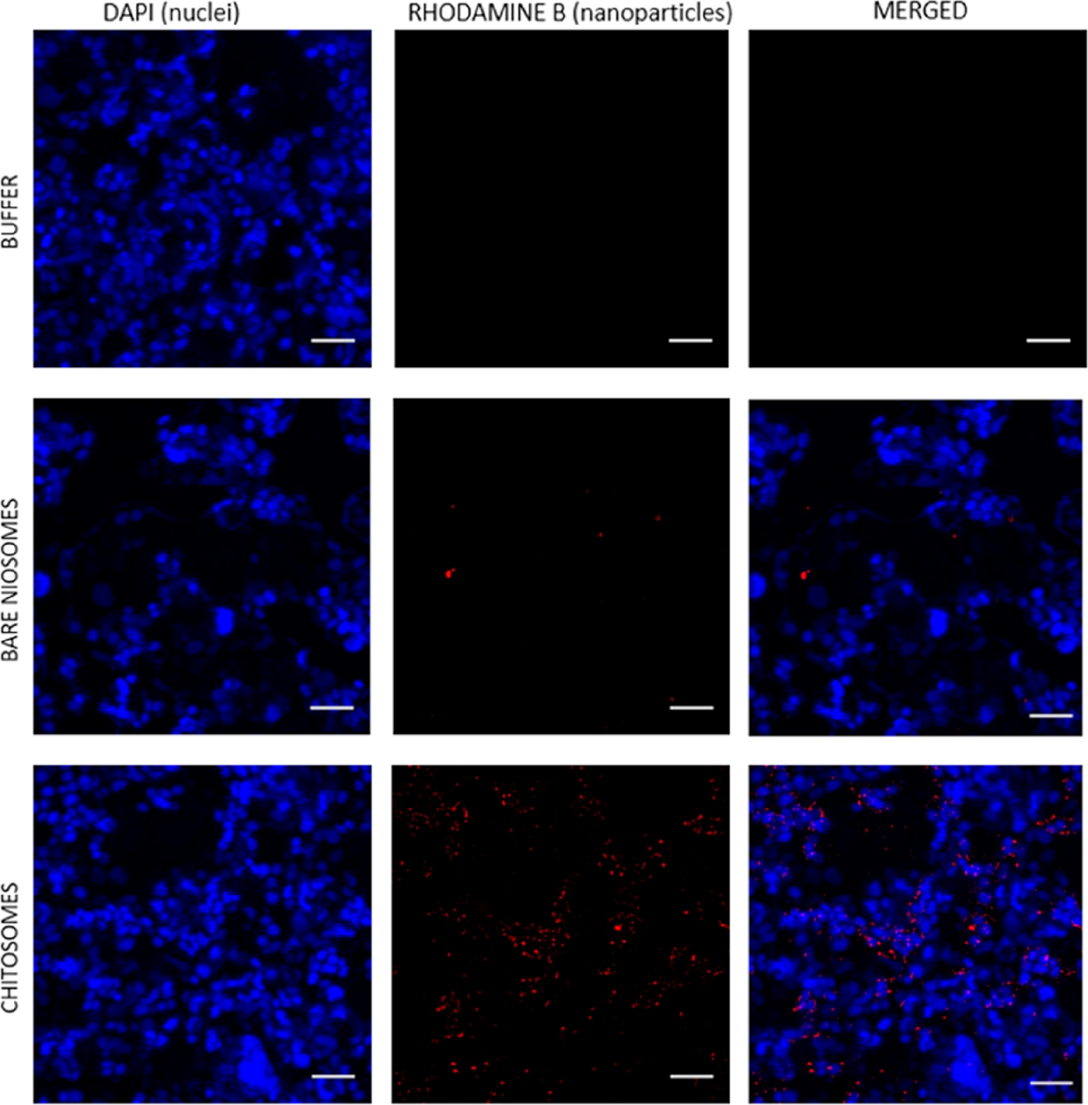
CLSM studies showing the interaction between niosomes or chitosomes with Caco-2/HT29-MTX cocultured cells (90:10 ratio). Cells were seeded in 8-chamber plates and allowed to attach and grow for 24 h. Then, the cells were washed with HBSS–HEPES, followed by incubation with niosomes or chitosomes at a concentration of 23.8 μg/mL in DMEM supplemented with 10 mM HEPES (pH 6.5) for 3 h. Cells incubated with DMEM supplemented with 10 mM of HEPES (pH 6.5) were used as control. Bare niosomes and chitosomes were labeled with Rhodamine B-DHPE (red), and cell nuclei were stained with DAPI (blue). The scale bars represent 50 μm.

The activation of the transcytosis pathway of Caco-2 for bare niosomes is already described in the literature.^19,23^ However, the coating of niosomes with chitosan could promote the adhesion and interaction with the cells of the GIT, which, in this study, was mimicked by using a Caco-2/HT29-MTX cell culture model. The interaction between fluorescently labeled niosomes or chitosomes with Caco-2/HT29-MTX cocultured cell monolayers was analyzed by CLSM (Figure 6). Results show that hardly any bare niosomes were found associated with the cells, while the number of chitosomes was clearly higher in the vicinity of the cells and presented a clearly higher level of interaction with the cellular monolayers. This is a consequence of the fact that chitosomes are coated with chitosan, which is positively charged and has mucoadhesive properties that facilitate electrostatic interactions and favor their adhesion to the surface of the negatively charged cells. This is also reflected in the SPR measurements with HT29-MTX cells, where it was seen that the SPR PAP response was evidently higher for chitosomes than for bare niosomes, which is caused by the stronger interaction and adherence of chitosomes to the cells (Figure S5). The CLSM results are also in good agreement with the interaction kinetics results obtained from the SPR measurements with HT29-MTX cells (Figure 5), which showed that the rate of the interaction of the chitosomes with a concentration of 23.8 μg/mL with the cell monolayer was higher than for the corresponding concentration of free chitosan.

**Figure 7:**
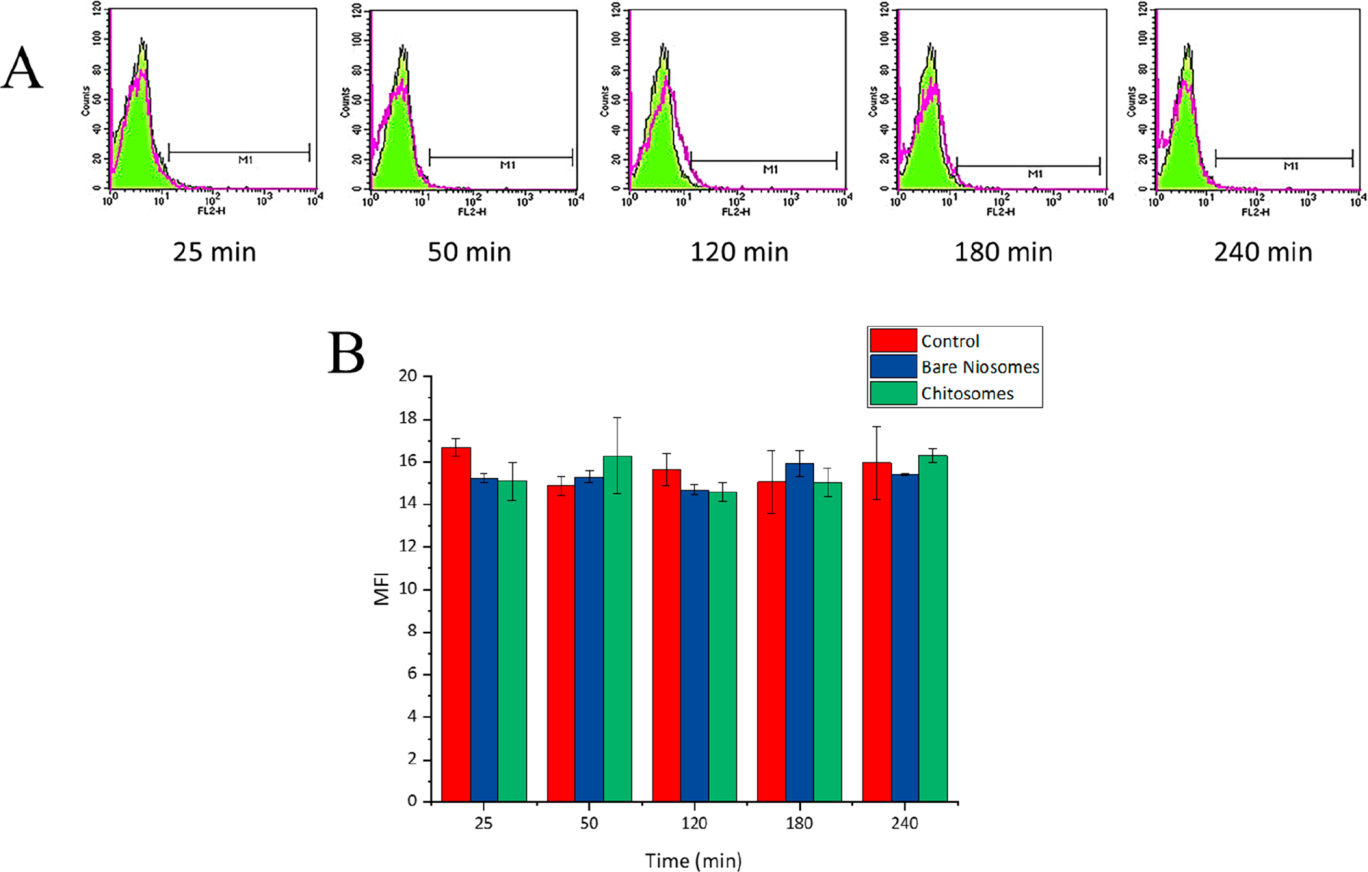
Intracellular uptake of Rhodamine B-labeled chitosomes in Caco-2/HT29-MTX cocultured cells studied by using flow cytometry analysis. Representative flow cytometry profiles of the intracellular uptake of chitosomes in Caco-2/HT29-MTX cocultured cells (A). The purple filled profile is control (negative cells), and green lines are cells treated with Rhodamine B-labeled chitosomes (positive cells) at the different time points (25, 50, 120, 180, and 240 min). MFI values of Caco-2/HT29-MTX cocultured cells treated with Rhodamine B-labeled chitosomes and the relative control (cells treated with cell culture medium pH 6.5) (B). Results are expressed as mean ± standard error of mean (SEM) of independent experiments.

Flow cytometry analysis was further performed to study the intracellular uptake of Rhodamine B-labeled chitosomes in cocultures of Caco-2/HT29-MTX cells. This was achieved by quenching the fluorescence of chitosomes associated or bound to the surface of the cells by treating the cells with trypan blue before the flow cytometry analysis. Caco-2/HT29-MTX cocultured cells were incubated with Rhodamine B-labeled chitosomes (23.8 μg/mL) or plain cell culture medium supplemented with 10 mM of HEPES (pH 6.5) (control) at different incubation times (25, 50, 120, 180, and 240 min). The flow cytometry profiles of one representative experiment are shown in Figure 7A. The internalization rate of fluorescent chitosomes was plotted as median fluorescence intensity (MFI) values (Figure 7B), which indicate the shift of intensity signal of fluorescent events counted after possible internalization of chitosomes by Caco-2/HT29-MTX cocultured cells and as relative positive events (Table S1). The MIF values did not show any differences at different incubation times. These results suggests that up to 240 min the chitosomes are not taken up by the cells and are mainly adsorbed strongly onto the surface of the cells.

Based on the flow cytometry results, it is apparent that the SPR responses and kinetics measured in this study during interaction of chitosomes with cell monolayers merely reflect the strength and ability of the chitosomes to interact with the cell surface, not the cell uptake efficacy, even though in earlier studies the SPR PAP responses have been shown to reflect also the cell uptake efficacy of other types of nanoparticles.^9,41−43^ Nonetheless, SPR measurements have their value in providing insights on the real-time interactions of nanoparticles with cells, especially as the measurements can be performed without the use of any labels that can affect the physicochemical properties of the nanoparticles of interest or affect cell behavior. Although it might be difficult to pinpoint the actual origin for the SPR PAP responses when measuring nanoparticle interactions with cells, i.e., cell surface interaction or cell uptake of nanoparticles, the SPR PAP responses and the rate constants extracted from the results can nevertheless be related to the strength of interaction or ability of the nanoparticles to interact with cells, which consequently reflects the drug delivery efficacy or targetability of the nanoparticles of interest.

Furthermore, even though chitosomes were not internalized by the Caco-2/HT29-MTX cocultured cell monolayer, as demonstrated by the confocal microscopy and flow cytometry measurements in this study, this does not mean that they could not be used as oral drug delivery systems. In fact, their tendency to strongly adhere to the cell surface instead of being internalized by cells can also be seen as an advantage as this would increase the retention time of chitosomes and allowing the release of possible drugs locally in the small intestine. Actually, it has been shown that, e.g., PLGA or PLLA nanoparticles that only adheres to cell surfaces, and which are not internalized by cells can deliver their payload to cells by extracellular drug release and/or direct drug transfer to contacting cells.^44,45^

## Conclusions

We have optimized and successfully prepared chitosomes as a potential nanoparticle-based system for oral administration. *In vitro* cell interaction studies with a unique cell-based SPR measuring platform using an intestinal cell model consisting of the mucus forming HT29-MTX cells revealed that chitosomes interact more efficiently with this cell monolayer than bare niosomes due to increased electrostatic interactions between chitosomes and the cells. Further SPR studies with other intestinal cell models of Caco-2 and Caco-2/HT29-MTX cell monolayers showed that the interaction kinetics for chitosomes and the intestinal cell models was faster in the presence of the mucus forming HT29-MTX cells, which is attributed to an increased electrostatic interaction and retention of chitosomes close to the cells due to the mucus forming HT29-MTX cells. These results were corroborated by confocal microscopy and flow cytometry studies but revealed that chitosomes were not actually taken up by cells in Caco-2/HT29-MTX cell monolayers. Thus, the cell-based SPR measuring platform proved to be a viable label-free tool to determine interaction kinetics between chitosomes and cell monolayers that reflected on their ability to interact with the cell surfaces in intestinal cell model layers.

## Supporting Information Available

Calibration curve of Tween 20 (Tw20); physicochemical characterization (size, PDI, and zeta-potential) of prescreening optimization of chitosomes and of different concentrations of niosomes and chitosomes in DMEM cell culture media; viability percentage of single HT29-MTX and Caco-2 cells after incubation with chitosomes at different concentrations and incubation times; SPR response for niosomes versus chitosomes with HT29-MTX monocultures; relative positive Caco-2/HT29-MTX cocultured cells treated with chitosomes (PDF)

## References

[ref1] Sun W.; Hu Q.; Ji W.; Wright G.; Gu Z. Leveraging Physiology for Precision Drug Delivery. Physiol Rev. 2017, 97, 189 10.1152/physrev.00015.2016.

[ref2] Patra J. K.; Das G.; Fraceto L. F.; Campos E. V. R.; del Pilar Rodriguez-Torres M.; Acosta-Torres L. S.; Diaz-Torres L. A.; Grillo R.; Swamy M. K.; Sharma S.; Habtemariam S.; Shin H.-S. Nano Based Drug Delivery Systems: Recent Developments and Future Prospects. J. Nanobiotechnology 2018, 10.1186/s12951-018-0392-8. PMC6145203 30231877

[ref3] Mailänder V.; Landfester K. Interaction of Nanoparticles with Cells. Biomacromolecules. 2009, 10, 2379 10.1021/bm900266r. 19637907

[ref4] Granqvist N.; Hanning A.; Eng L.; Tuppurainen J.; Viitala T. Label-Enhanced Surface Plasmon Resonance: A New Concept for Improved Performance in Optical Biosensor Analysis. Sensors (Switzerland) 2013, 13, 15348 10.3390/s131115348. PMC3871110 24217357

[ref5] Nguyen H. H.; Park J.; Kang S.; Kim M. Surface Plasmon Resonance: A Versatile Technique for Biosensor Applications. Sensors (Switzerland). 2015, 15, 10481 10.3390/s150510481. PMC4481982 25951336

[ref6] Liu Y.; Daum P. H. Relationship of Refractive Index to Mass Density and Self-Consistency of Mixing Rules for Multicomponent Mixtures like Ambient Aerosols. J. Aerosol Sci. 2008, 39, 974 10.1016/j.jaerosci.2008.06.006.

[ref7] Korhonen K.; Granqvist N.; Ketolainen J.; Laitinen R. Monitoring of Drug Release Kinetics from Thin Polymer Films by Multi-Parametric Surface Plasmon Resonance. Int. J. Pharm. 2015, 494 (1), 531–536. 10.1016/j.ijpharm.2015.08.071. 26319634

[ref8] Viitala T.; Granqvist N.; Hallila S.; Raviña M.; Yliperttula M. Elucidating the Signal Responses of Multi-Parametric Surface Plasmon Resonance Living Cell Sensing: A Comparison between Optical Modeling and Drug-MDCKII Cell Interaction Measurements. PLoS One 2013, 8 (8), e72192 10.1371/journal.pone.0072192. 24015218 PMC3754984

[ref9] Suutari T.; Silen T.; SŞen Karaman D.; Saari H.; Desai D.; Kerkelä E.; Laitinen S.; Hanzlikova M.; Rosenholm J. M.; Yliperttula M.; Viitala T. Real-Time Label-Free Monitoring of Nanoparticle Cell Uptake. Small 2016, 12, 6289 10.1002/smll.201601815. 27690329

[ref10] Kari O. K.; Ndika J.; Parkkila P.; Louna A.; Lajunen T.; Puustinen A.; Viitala T.; Alenius H.; Urtti A. In Situ Analysis of Liposome Hard and Soft Protein Corona Structure and Composition in a Single Label-Free Workflow. Nanoscale 2020, 12 (3), 1728–1741. 10.1039/C9NR08186K. 31894806

[ref11] Tavakoli S.; Kari O. K.; Turunen T.; Lajunen T.; Schmitt M.; Lehtinen J.; Tasaka F.; Parkkila P.; Ndika J.; Viitala T.; Alenius H.; Urtti A.; Subrizi A. Diffusion and Protein Corona Formation of Lipid-Based Nanoparticles in the Vitreous Humor: Profiling and Pharmacokinetic Considerations. Mol. Pharmacol. 2021, 18, 699 10.1021/acs.molpharmaceut.0c00411. PMC7856631 32584047

[ref12] Mali A. D.; Bathe R. S. An Updated Review on Liposome Drug Delivery System. Asian Journal of Pharmaceutical Research 2015, 5, 151 10.5958/2231-5691.2015.00023.4.

[ref13] He H.; Lu Y.; Qi J.; Zhu Q.; Chen Z.; Wu W. Adapting Liposomes for Oral Drug Delivery. Acta Pharmaceutica Sinica B 2019, 9, 36 10.1016/j.apsb.2018.06.005. 30766776 PMC6362257

[ref14] Louis D. An Overview on Niosomes: A Drug Nanocarrier. Drug Designing & Intellectual Properties International Journal 2018, 1 (5), 143–151. 10.32474/DDIPIJ.2018.01.000125.

[ref15] Marianecci C.; di Marzio L.; Rinaldi F.; Celia C.; Paolino D.; Alhaique F.; Esposito S.; Carafa M. Niosomes from 80s to Present: The State of the Art. Adv. Colloid Interface Sci. 2014, 205, 187 10.1016/j.cis.2013.11.018. 24369107

[ref16] Primavera R.; Palumbo P.; Celia C.; Cinque B.; Carata E.; Carafa M.; Paolino D.; Cifone M. G.; di Marzio L. An Insight of in Vitro Transport of PEGylated Non-Ionic Surfactant Vesicles (NSVs) across the Intestinal Polarized Enterocyte Monolayers. Eur. J. Pharm. Biopharm. 2018, 127, 432–442. 10.1016/j.ejpb.2018.03.013. 29605467

[ref17] Sandri G.; Rossi S.; Bonferoni M. C.; Ferrari F.; Mori M.; Caramella C. The Role of Chitosan as a Mucoadhesive Agent in Mucosal Drug Delivery. Journal of Drug Delivery Science and Technology. 2012, 22, 275 10.1016/S1773-2247(12)50046-8.

[ref18] Sogias I. A.; Williams A. C.; Khutoryanskiy V. V. Why Is Chitosan Mucoadhesive?. Biomacromolecules 2008, 9, 1837 10.1021/bm800276d. 18540644

[ref19] Primavera R.; Palumbo P.; Celia C.; Cilurzo F.; Cinque B.; Carata E.; Carafa M.; Paolino D.; Cifone M. G.; di Marzio L. Corrigendum to “An Insight of in Vitro Transport of PEGylated Non-Ionic Surfactant Vesicles (NSVs) across the Intestinal Polarized Enterocyte Monolayers” [Eur. J. Pharm. Biopharm. 127 (2018) 432–442] (S0939641117315114) (10.1016/j.Ejpb.2018.03.013)). European Journal of Pharmaceutics and Biopharmaceutics. 2018, 128, 259 10.1016/j.ejpb.2018.05.010. 29605467

[ref20] Cristiano M. C.; Cosco D.; Celia C.; Tudose A.; Mare R.; Paolino D.; Fresta M. Anticancer Activity of All-Trans Retinoic Acid-Loaded Liposomes on Human Thyroid Carcinoma Cells. Colloids Surf. B Biointerfaces 2017, 150, 408 10.1016/j.colsurfb.2016.10.052. 27829536

[ref21] Torrieri G.; Fontana F.; Figueiredo P.; Liu Z.; Ferreira M. P. A.; Talman V.; Martins J. P.; Fusciello M.; Moslova K.; Teesalu T.; Cerullo V.; Hirvonen J.; Ruskoaho H.; Balasubramanian V.; Santos H. A. Dual-Peptide Functionalized Acetalated Dextran-Based Nanoparticles for Sequential Targeting of Macrophages during Myocardial Infarction. Nanoscale 2020, 12, 2350 10.1039/C9NR09934D. 31930241

[ref22] Martins J. P.; Liu D.; Fontana F.; Ferreira M. P. A.; Correia A.; Valentino S.; Kemell M.; Moslova K.; Mäkilä E.; Salonen J.; Hirvonen J.; Sarmento B.; Santos H. A. Microfluidic Nanoassembly of Bioengineered Chitosan-Modified FcRn-Targeted Porous Silicon Nanoparticles @ Hypromellose Acetate Succinate for Oral Delivery of Antidiabetic Peptides. ACS Appl. Mater. Interfaces 2018, 10, 44354 10.1021/acsami.8b20821. 30525379

[ref23] Primavera R.; Palumbo P.; Celia C.; Cinque B.; Carata E.; Carafa M.; Paolino D.; Cifone M. G.; di Marzio L. An Insight of in Vitro Transport of PEGylated Non-Ionic Surfactant Vesicles (NSVs) across the Intestinal Polarized Enterocyte Monolayers. Eur. J. Pharm. Biopharm. 2018, 127, 432 10.1016/j.ejpb.2018.03.013. 29605467

[ref24] di Marzio L.; Marianecci C.; Cinque B.; Nazzarri M.; Cimini A. M.; Cristiano L.; Cifone M. G.; Alhaique F.; Carafa M. PH-Sensitive Non-Phospholipid Vesicle and Macrophage-like Cells: Binding, Uptake and Endocytotic Pathway. Biochim Biophys Acta Biomembr. 2008, 1778, 2749 10.1016/j.bbamem.2008.07.029. 18762164

[ref25] Dormidontova E. E. Role of Competitive PEO-Water and Water-Water Hydrogen Bonding in Aqueous Solution PEO Behavior. Macromolecules 2002, 35, 987 10.1021/ma010804e.

[ref26] Fathi Azarbayjani A.; Tan E. H.; Chan Y. W.; Chan S. Y. Transdermal Delivery of Haloperidol by Proniosomal Formulations with Non-Ionic Surfactants. Biol. Pharm. Bull. 2009, 32, 1453 10.1248/bpb.32.1453. 19652389

[ref27] Kamboj S.; Saini V.; Bala S. Formulation and Characterization of Drug Loaded Nonionic Surfactant Vesicles (Niosomes) for Oral Bioavailability Enhancement. Scientific World Journal 2014, 2014, 1 10.1155/2014/959741. PMC3929577 24672401

[ref28] Philippova O. E.; Volkov E. v.; Sitnikova N. L.; Khokhlov A. R.; Desbrieres J.; Rinaudo M. Two Types of Hydrophobic Aggregates in Aqueous Solutions of Chitosan and Its Hydrophobic Derivative. Biomacromolecules 2001, 2, 483 10.1021/bm005649a. 11749210

[ref29] Shilova S. v.; Tret’yakova A. Y.; Barabanov V. P. Association of Chitosan in Aqueous-Alcohol Solutions. Polymer Science - Series A 2018, 60, 184 10.1134/S0965545X1802013X.

[ref30] Yanagisawa M.; Kato Y.; Yoshida Y.; Isogai A. SEC-MALS Study on Aggregates of Chitosan Molecules in Aqueous Solvents: Influence of Residual N-Acetyl Groups. Carbohydr. Polym. 2006, 66, 192 10.1016/j.carbpol.2006.03.008.

[ref31] Wang Q. Z.; Chen X. G.; Liu N.; Wang S. X.; Liu C. S.; Meng X. H.; Liu C. G. Protonation Constants of Chitosan with Different Molecular Weight and Degree of Deacetylation. Carbohydr. Polym. 2006, 65, 194 10.1016/j.carbpol.2006.01.001.

[ref32] di Marzio L.; Esposito S.; Rinaldi F.; Marianecci C.; Carafa M. Polysorbate 20 Vesicles as Oral Delivery System: In Vitro Characterization. Colloids Surf. B Biointerfaces 2013, 104, 200–206. 10.1016/j.colsurfb.2012.10.036. 23314494

[ref33] di Marzio L.; Marianecci C.; Petrone M.; Rinaldi F.; Carafa M. Novel PH-Sensitive Non-Ionic Surfactant Vesicles: Comparison between Tween 21 and Tween 20. Colloids Surf. B Biointerfaces 2011, 82, 18 10.1016/j.colsurfb.2010.08.004. 20832262

[ref34] di Marzio L.; Esposito S.; Rinaldi F.; Marianecci C.; Carafa M. Polysorbate 20 Vesicles as Oral Delivery System: In Vitro Characterization. Colloids Surf. B Biointerfaces 2013, 104, 200–206. 10.1016/j.colsurfb.2012.10.036. 23314494

[ref35] Martins J. P.; Figueiredo P.; Wang S.; Espo E.; Celi E.; Martins B.; Kemell M.; Moslova K.; Mäkilä E.; Salonen J.; Kostiainen M. A.; Celia C.; Cerullo V.; Viitala T.; Sarmento B.; Hirvonen J.; Santos H. A. Neonatal Fc Receptor-Targeted Lignin-Encapsulated Porous Silicon Nanoparticles for Enhanced Cellular Interactions and Insulin Permeation across the Intestinal Epithelium. Bioact Mater. 2022, 9, 299–315. 10.1016/j.bioactmat.2021.08.007. 34820572 PMC8586719

[ref36] Moore T. L.; Rodriguez-Lorenzo L.; Hirsch V.; Balog S.; Urban D.; Jud C.; Rothen-Rutishauser B.; Lattuada M.; Petri-Fink A. Nanoparticle Colloidal Stability in Cell Culture Media and Impact on Cellular Interactions. Chem. Soc. Rev. 2015, 44 (17), 6287–6305. 10.1039/C4CS00487F. 26056687

[ref37] Frigaard J.; Jensen J. L.; Galtung H. K.; Hiorth M. The Potential of Chitosan in Nanomedicine: An Overview of the Cytotoxicity of Chitosan Based Nanoparticles. Front Pharmacol 2022, 13 (May), 1–19. 10.3389/fphar.2022.880377. PMC9115560 35600854

[ref38] Oransa H. A.; Boughdady M. F. Novel Mucoadhesive Chitosomes as a Platform for Enhanced Oral Bioavailability of Cinnarizine. Int. J. Nanomed. 2022, 17, 5641–5660. 10.2147/IJN.S384494. PMC9704018 36452306

[ref39] Sahuri-arisoylu M.; Mould R. R.; Shinjyo N.; Bligh S. W. A.; Nunn A. V. W.; Guy G. W.; Thomas E. L.; Bell J. D. Acetate Induces Growth Arrest in Colon Cancer Cells Through Modulation of Mitochondrial Function. Frontiers in Nutrition 2021, 8 (April), 1–10. 10.3389/fnut.2021.588466. PMC8081909 33937302

[ref40] Béduneau A.; Tempesta C.; Fimbel S.; Pellequer Y.; Jannin V.; Demarne F.; Lamprecht A. A Tunable Caco-2/HT29-MTX Co-Culture Model Mimicking Variable Permeabilities of the Human Intestine Obtained by an Original Seeding Procedure. Eur. J. Pharm. Biopharm. 2014, 87, 290 10.1016/j.ejpb.2014.03.017. 24704198

[ref41] Kauppila M.; Ståhlberg R.; Francisco V.; Ferreira L.; Skottman H. Multi-parametric Surface Plasmon Resonance-based Intake Quantification of Label-free Light-activated Nanoparticles by Therapeutic Limbal Stem Cells for Corneal Blindness. Nano Select 2022, 3 (8), 1232–1241. 10.1002/nano.202200027.

[ref42] Koponen A.; Kerkelä E.; Rojalin T.; Lázaro-Ibáñez E.; Suutari T.; Saari H. O.; Siljander P.; Yliperttula M.; Laitinen S.; Viitala T. Label-Free Characterization and Real-Time Monitoring of Cell Uptake of Extracellular Vesicles. Biosens Bioelectron 2020, 168, 112510 10.1016/j.bios.2020.112510. 32877783

[ref43] Akl M. A.; Kartal-Hodzic A.; Suutari T.; Oksanen T.; Montagner I. M.; Rosato A.; Ismael H. R.; Afouna M. I.; Caliceti P.; Yliperttula M.; Samy A. M.; Mastrotto F.; Salmaso S.; Viitala T. Real-Time Label-Free Targeting Assessment and in Vitro Characterization of Curcumin-Loaded Poly-Lactic-Co-Glycolic Acid Nanoparticles for Oral Colon Targeting. ACS Omega 2019, 4 (16), 16878–16890. 10.1021/acsomega.9b02086. 31646234 PMC6796886

[ref44] Hofmann D.; Messerschmidt C.; Bannwarth M. B.; Landfester K.; Mailänder V. Drug Delivery without Nanoparticle Uptake: Delivery by a Kiss-and-Run Mechanism on the Cell Membrane. Chem. Commun. 2014, 50 (11), 1369–1371. 10.1039/C3CC48130A. 24346146

[ref45] Xu P.; Gullotti E.; Tong L.; Highley C. B.; Errabelli D. R.; Hasan T.; Cheng J.-X.; Kohane D. S.; Yeo Y. Intracellular Drug Delivery by Poly(Lactic-Co-Glycolic Acid) Nanoparticles. Mol. Pharmaceutics 2009, 6 (1), 190–201. 10.1021/mp800137z. PMC2653259 19035785

