# Supplementary material for: In Vitro Characterization and Real-Time Label-Free Assessment of the Interaction of Chitosan-Coated Niosomes with Intestinal Cellular Monolayers
Source: Langmuir. 2023 Jun 2;39(23):8255–66. doi: 10.1021/acs.langmuir.3c00728 (PMC10269435; doi:10.1021/acs.langmuir.3c00728)
Supplement: Supplementary file 1 — la3c00728_si_001.pdf [file la3c00728_si_001.pdf]

Supporting information

In vitro characterization and real-time label-free assessment of the interaction of chitosan-coated niosomes with intestinal cellular monolayers

Elena Scurti[#,$], João Pedro Martins[#], Christian Celia[$,||], Paola Palumbo[§], Francesca Lombardi[§], Dalila Iannotta[$,£], Luisa Di Marzio[$,*], Hélder A. Santos[#,!!,‡,*], Tapani Viitala[#,¤,*]

[#]Drug Research Program, Division of Pharmaceutical Chemistry and Technology, Faculty of Pharmacy, University of Helsinki, Helsinki FI-00014, Finland

[$]Department of Pharmacy, University of Chieti – Pescara "G. d'Annunzio", Chieti I-66100, Italy.

[||]Laboratory of Drug Targets Histopathology, Institute of Cardiology, Lithuanian University of Health Sciences, A. Mickeviciaus g. 9, Kaunas LT-44307, Lithuania.

[§]Department of Life, Health & Environmental Sciences, University of L'Aquila, L'Aquila I-67100, Italy.

[£]Australian Institute for Bioengineering and Nanotechnology, The University of Queensland, Brisbane QLD 4072, Australia.

[!!]Department of Biomedical Engineering, University Medical Center Groningen, University of Groningen, Ant. Deusinglaan 1, Groningen, 9713 AV, The Netherlands.

‡W.J. Kolff Institute for Biomedical Engineering and Materials Science, University Medical Center Groningen, University of Groningen, Ant. Deusinglaan 1, Groningen, 9713 AV, The Netherlands.

¤Pharmaceutical Sciences Laboratory, Faculty of Science and Engineering, Åbo Akademi University, Turku FI-20520, Finland

*Corresponding authors: Luisa Di Marzio (L.D.M.): luisa.dimarzio@unich.it; Hélder A. Santos (H.A.S.): h.a.santos@umcg.nl; Tapani Viitala (T.V.): tapani.viitala@helsinki.fi

Supplementary Figures

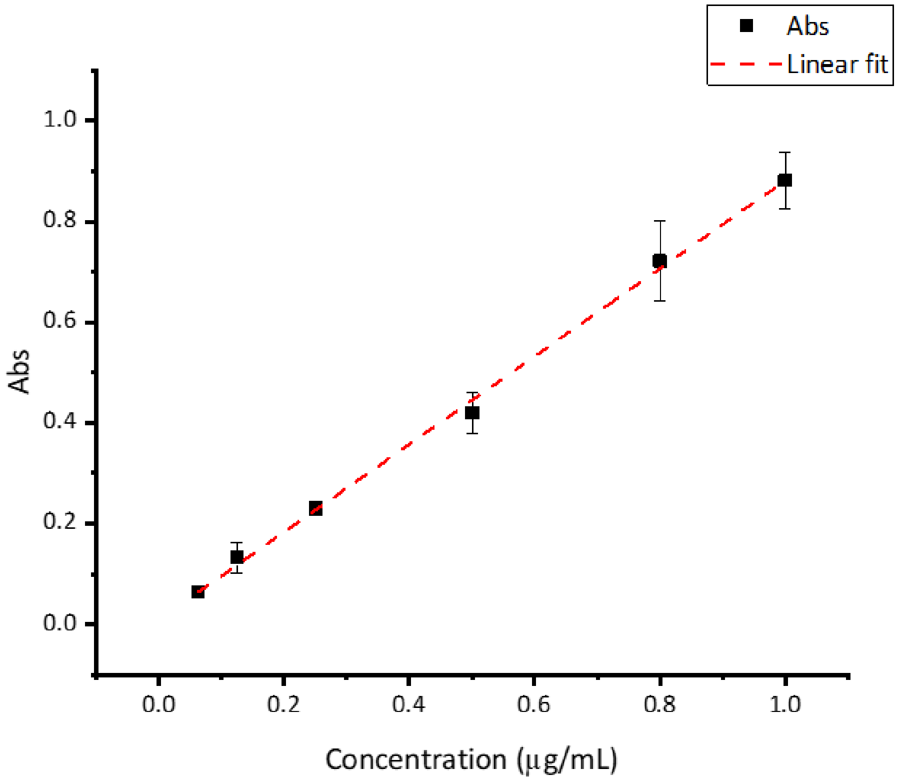

Figure S1. Calibration curve for Tween® 20. The calibration curve was used to assess the percentage of self-assembly of the surfactant to obtain niosomes. Average values are the result of six independent measurements ± standard deviation (S.D). Trendline equation: $y = 0.8725x + 0.0098$; $R^2 = 0.998$.

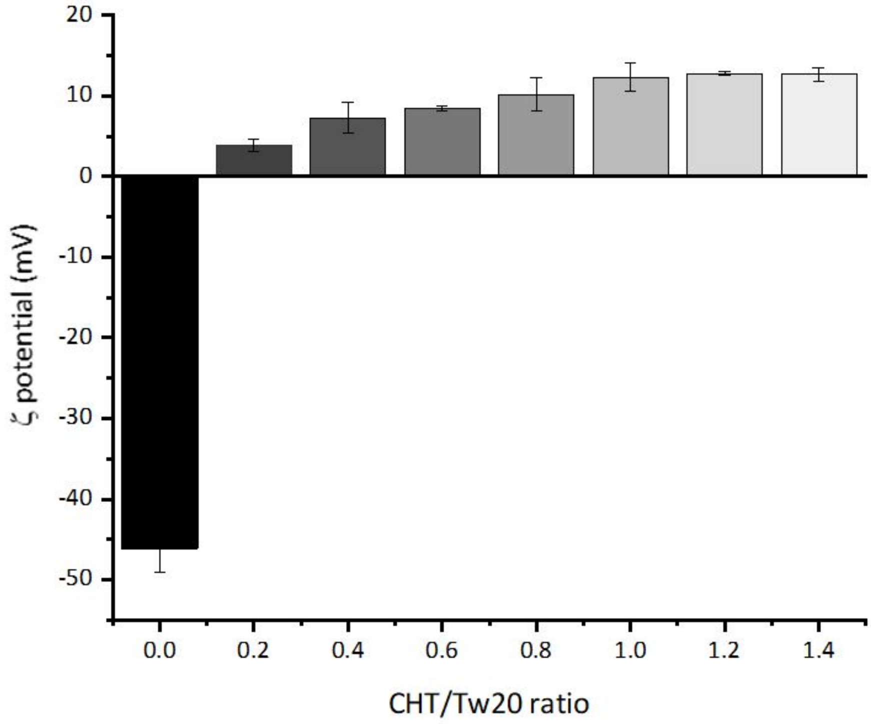

Figure S2. Pre-screening for optimization of chitosome synthesis. Zeta (ζ)-potential of native, undiluted samples of bare niosomes (black bar) and chitosomes using different ratios of chitosan/Tween® 20 (w/w) (grey scale bars; the lighter the grey color of the bar is, the larger the chitosan/Tween® 20 ratio). Results are presented as mean ± standard deviation (S.D.) ($n = 3$).

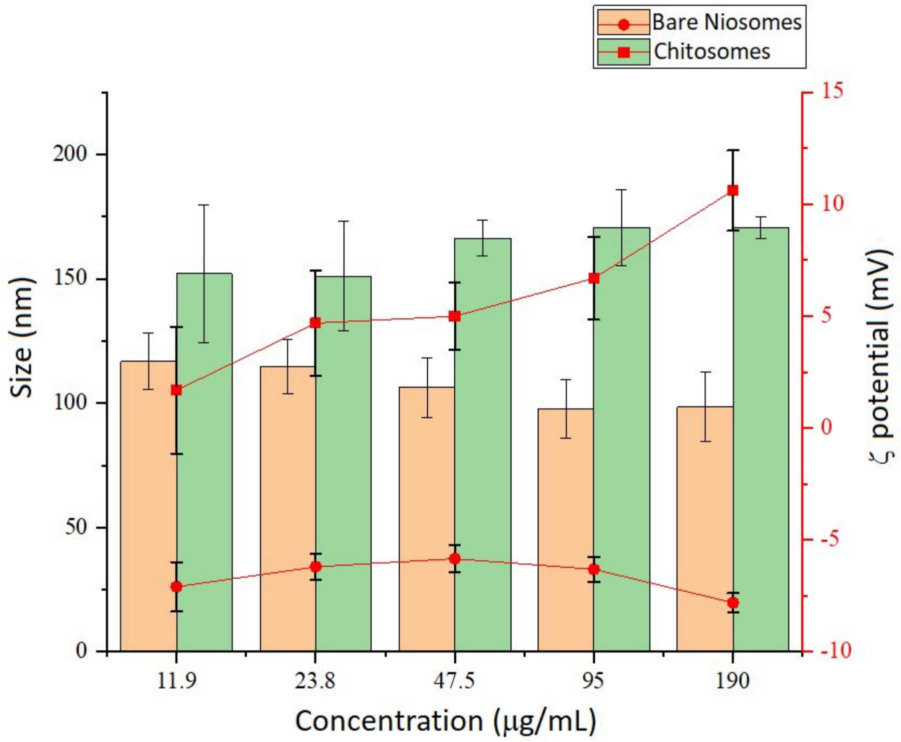

Figure S3. Size and ζ-potential measurements for different concentrations of niosomes and chitosomes in DMEM cell culture media supplemented with 10 mM of HEPES (pH 6.5). Results are representative of three independent experiments ± standard deviation (S.D.).

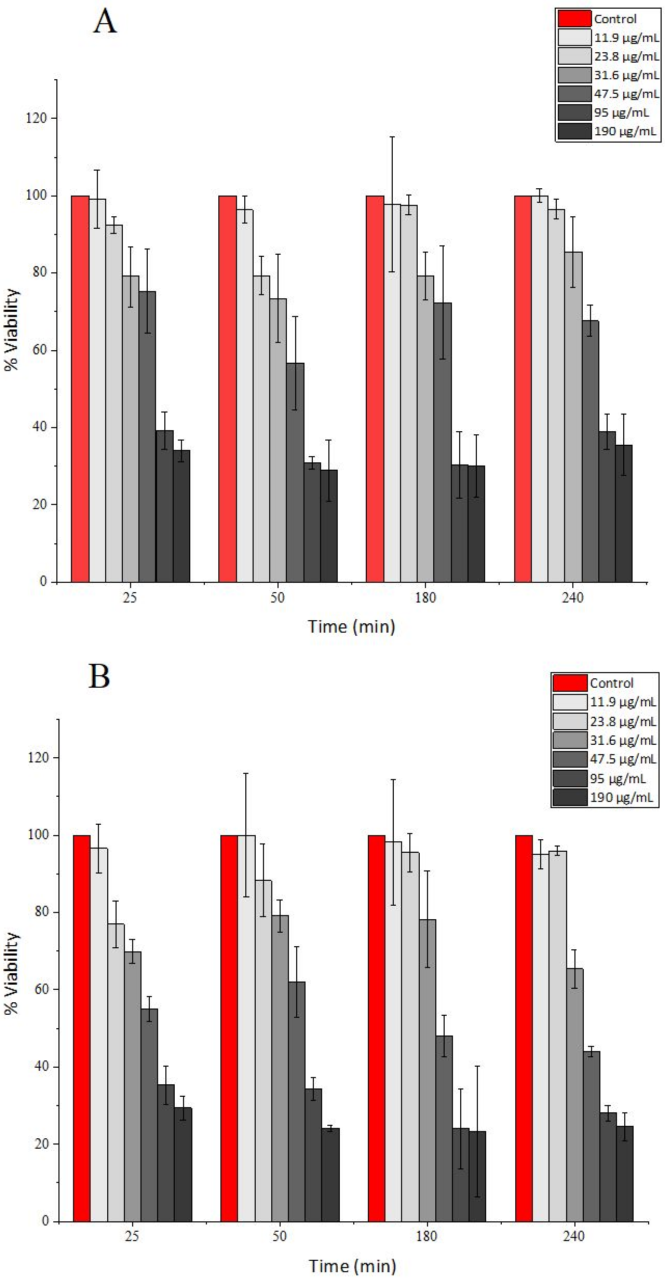

sc

Figure S4. Viability of HT29-MTX (A) and Caco-2 (B) monocultures treated with chitosomes at concentrations ranging from 11.9 µg/mL to 190 µg/mL, and different incubation times (25–240 min). Data was compared to control consisting of cell culturing media at pH 6.5 (supplemented with 10 mM of HEPES). Results are presented as mean ± S.D. (n = 3).

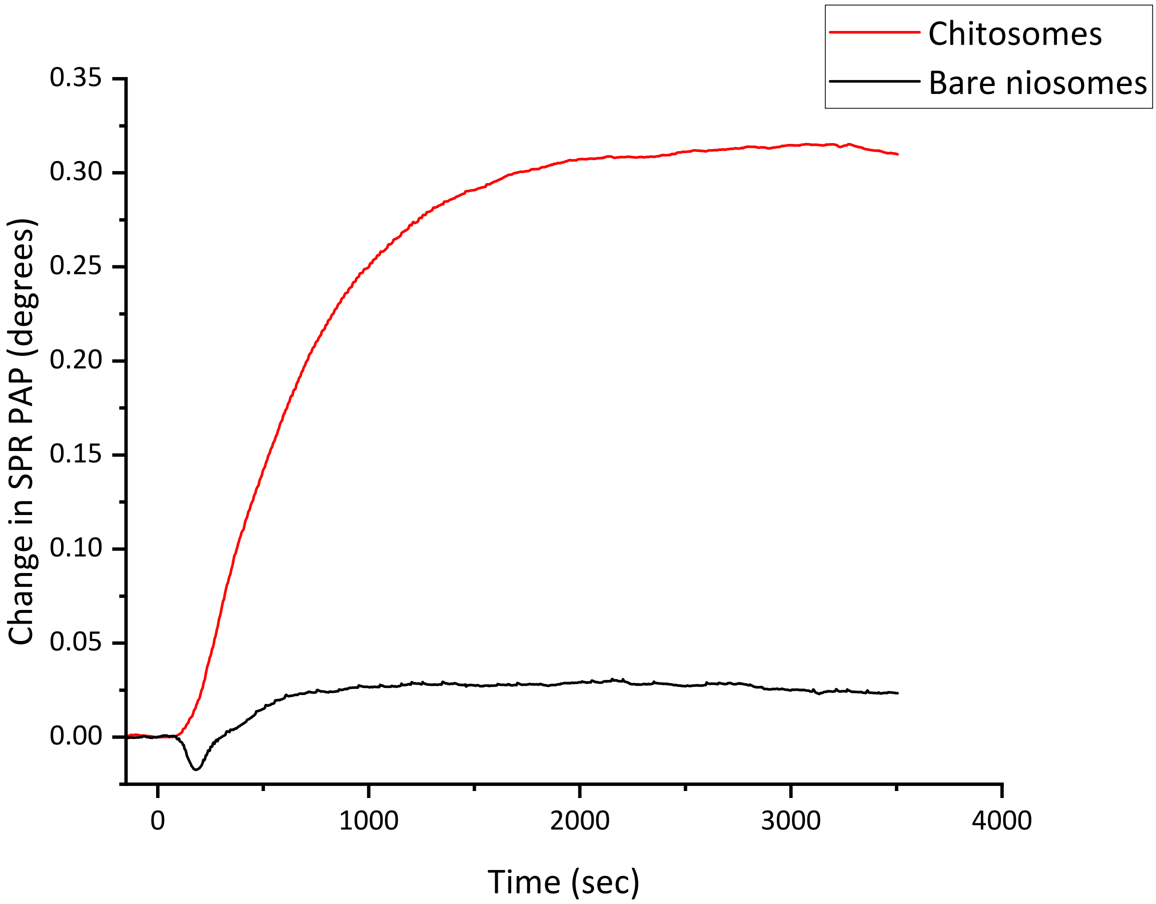

Figure S5. SPR PAP variation of the interaction of niosomes (black) and chitosomes (red) with HT29-MTX monocultures. The samples were diluted 1:20 (v/v) to a final concentration of 47.5 μg/mL from the stock solution (0.95 mg/mL) in SPR buffer.

Table S1. Percentage of positive Caco-2/HT29-MTX co-cultured cells treated with Rhodamine B-labeled chitosomes (23.8 μg/mL) detected by using flow cytometry analysis. Results were expressed as mean ± Standard Error of Mean (SEM) of two independent experiments in duplicates.

**Time (min)**	**25**	**50**	**120**	**180**	**240**
**Positive cells (%)**	3.00 ± 0.76	1.90 ± 0.21	8.70 ± 1.84	4.08 ± 0.64	1.82 ± 0.09
